# Prepare for slingshot

**DOI:** 10.1172/jci.insight.203333

**Published:** 2026-01-09

**Authors:** Oliver Eickelberg

There are few aspects of my profession that I enjoy as much as reading, evaluating, and discussing primary research papers. Hence, I was honored and excited to assume the role of Editor in Chief of *JCI Insight* in September 2024. With only slightly more than 15 months on the job, it is my honor and pleasure to contribute this Editorial for the ten-year anniversary of *JCI Insight*. It is safe to say that, as a journal, we are now entering adolescence.

I prepared this Editorial as we were celebrating the Thanksgiving holiday, so bear with me as I express my sincere gratitude for the past 15 months at *JCI Insight* to the following people: First and foremost, to Corinne Williams, our Senior Science Editor, without whom this Journal would hardly thrive. Second, to Sarah Jackson, Executive Editor of the JCI Family of Journals, who masterfully coordinates and oversees all publishing activities. Third, to our wonderful and expert team of Deputy and Associate Editors at the University of Pittsburgh — Yinz rock! Fourth, to the ASCI, its leadership, and *JCI Insight* Founding Editor Howard Rockman for delivering on the bold vision to establish *JCI Insight* as a sister journal to the *Journal of Clinical Investigation* (*JCI*) back in 2016. What a courageous feat to lead two journals that are self-published by the ASCI and establish the founding vision for this Journal by publishing “preclinical, translational, and clinical research that uncovers new insights into the basis of disease and therapeutic approaches” (1). To Kathleen Collins, the immediate past *JCI Insight* Editor in Chief, and her team at the University of Michigan, for steering the Journal through the COVID-19 pandemic and establishing a range of new initiatives, particularly the Physician-Scientist Development section. To Elizabeth McNally and the entire *JCI* editorial team at Northwestern University for providing invaluable insight and partnership for *JCI Insight* submissions coming from the Dual-Journal Submission track or as transfers from the *JCI*. To my institution and its leadership, in particular Dean Anantha Shekhar and Chair of Medicine Anne Marie Lennon, for generously supporting the Editorial Board and me for this tenure. These are rocky times for institutional support mechanisms for scientific ventures like this; hence, your support is even more appreciated. And finally, to our academic community — of authors, reviewers, and readers — who donate precious time and insight for a service so essential to the mission of our academic institutions to better understand, detect, prevent, and cure disease.

Here we are, a decade later, with over 4,500 articles published and more than 150,000 citations, and a reader- and authorship that spans almost every continent. So where do we steer our spacecraft from here over the next years? Our current University of Pittsburgh–based team onboarded about a year ago with the mission “Go, Flight,” trusting in successful liftoff (2). I would argue that we have successfully launched and established cruising altitude and speed for steady space flight, avoiding asteroid debris where possible. We now aim for our first slingshot maneuver acceleration soon. In the cargo bay of our spacecraft, we have successfully loaded several new initiatives, including the recently established DualJournal Submission track in collaboration with the *JCI*; thematic Journal issues (the first of which is on cross-organ fibrosis, to be published mid-2026), an expanded Physician-Scientist Development section; regularly featured state-of-the-art reviews; onboarding of early-stage investigators (ASCI/Young Physician Scientist Award [YPSA] and Emerging-Generation [E-Gen] Award recipients) to the Editorial Board; and inclusion of YPSA awardees in our guaranteed external review program. *JCI Insight* is proud to support physician-scientists throughout all stages of their career, with an emphasis on those in the early stages.

As we look to the next decade of *JCI Insight*, we will continuously aim to improve upon aspects of scientific publishing that we can and should do better on. The peer review process remains under scrutiny. Our scientific community, as well as the general public, questions whether peer review will and should remain the gold-standard process to evaluate scientific findings. Many question whether we should abandon this laborious and time-consuming process and instead rapidly publish all scientific literature on freely accessible servers for everyone, without peer-review or editorial processing. I would argue that we absolutely need to maintain and protect free and open access of federally funded (i.e., taxpayer-supported) biomedical research, a process fully supported at the *JCI* and *JCI Insight*. I would also argue, however, against abandoning peer review entirely and switching to postpublication discussions and evaluations instead. For example, during the initial months of the COVID-19 pandemic, non-peer-reviewed preprint publications were rapidly disseminated and expeditiously amplified by media outlets, which undoubtedly led to confusing and harmful consequences and ethically questionable actions for our patients. This is only one but an important advantage of the peer review publication process.

At *JCI Insight*, we continue to strive toward a best-practices scientific publication platform that provides expedited yet balanced and fair peer review and rapid dissemination of results from biomedical scientific research to the specialized and general public. We seek to continuously refine and improve our process for the benefit of all our authors and readers to publish scientifically exciting and rigorous work. Do we always get it right? Do we process every manuscript in the most expedited fashion? Likely not, but we certainly strive to do so. Let me take you through our process for any a new submission we receive: Each new submission is carefully evaluated for scientific novelty, rigor, and clarity of conclusions, among other criteria, before being sent for outside review. A team of two to three Editors decides on editorial rejections for submissions that exhibit major shortcomings, e.g., lack of rigor or novelty. Papers that are sent out for external review are often presented to and discussed by the entire Editorial Board at its weekly meetings. Manuscripts that are on track for publication undergo comprehensive quality control checks from a team of scientific editors, copy editors, and production editors. Thus, from submission to ultimate publication, each article has been meticulously evaluated and edited by a number of scientists and editors. In addition, rigorous image analysis is performed prior to acceptance, a process that involves both image screening software and human eyes (3). In our brief tenure, we have had to contact authors 46 times about images that raised concerns and have declined to move forward with 5 manuscripts in such cases.

One of the most rewarding experiences over the past year has been the positive feedback from our authors on transparency and communication with the editorial team in case of questions. Many authors have voiced that our peer review process has scientifically improved their submission, irrespective of whether we or others have published it. These benefits to both authors and the scientific community are why we do what we do. We love receiving positive feedback from our authors, reviewers, and readers — Please keep sending it. We will also entertain your grievances and hope to further refine our processes based on the above.

Without a doubt, several challenges to our core mission are in our flight path, and we need to prepare for potential impact. Current funding levels for fundamental biomedical research remain vulnerable in many countries. The true extent of the reproducibility crisis remains unknown and will likely not easily or magically resolve. The tidal wave of AI-generated submissions, peer reviews, and correspondence threatens to devalue the impact of the individual human investigator. At the same time, our opportunities are undoubtedly breathtaking: The scientific community currently enjoys access to open datasets of unprecedented size and includes a generation of researchers who have been trained using open-source databases and open-access publications. These scientists see data sharing as the default approach in science, taking advantage of a wealth of knowledge that was unimaginable even just ten years ago.

To our authors, reviewers, readers, and staff — past, present, and future — who made and will continue to make this possible: Thank you. Many of your submissions and reviews are scientific masterpieces that form the heart of this Journal. The next ten years will demand even more imagination, even more collaboration, even more courage. In this spirit, cheers to the next ten years of *JCI Insight*!

## Version 1 01/09/2026

Electronic publication
